# Assessing regional variations in the effect of the removal of user fees on facility-based deliveries in rural Zambia

**DOI:** 10.4314/ahs.v17i4.28

**Published:** 2017-12

**Authors:** Chama-Chiliba Chitalu, Koch Steven

**Affiliations:** 1 University of Zambia, Department of Economics; University of Pretoria, Department of Economics; 2 University of Pretoria, Department of Economics

**Keywords:** Maternal care, facility based deliveries, user fees, rural, Zambia

## Abstract

**Background:**

Maternal health remains a concern in sub-Saharan Africa, where maternal mortality averages 680 per 100,000 live births and almost 50% of the approximately 350,000 annual maternal deaths occur. Improving access to skilled birth assistance is paramount to reducing this average, and user fee reductions could help.

**Objective:**

The aim of this research was to analyse the effect of user fee removal in rural areas of Zambia on the use of health facilities for childbirth. The analysis incorporates supply-side factors, including quantitative measures of service quality in the assessment.

**Method:**

The analysis uses quarterly longitudinal data covering 2003 (q1)-2008 (q4) and controls for unobserved heterogeneity, spatial dependence and quantitative supply-side factors within an Interrupted Time Series design.

**Results:**

User fee removal was found to initially increase aggregate facility-based deliveries. Drug availability, the presence of traditional birth attendants, social factors and cultural factors also influenced facility-based deliveries at the national level.

**Conclusion:**

Although user fees matter, to a degree, service quality is a relatively more important contributor to the promotion of facility-based deliveries. Thus, in the short-term, strengthening and improving community-based interventions could lead to further increases in facility-based deliveries.

## Introduction

Maternal health remains a global challenge in sub-Saharan Africa, where maternal mortality averages 680 per 100,000 live births and almost 50% of the approximately 350,000 annual maternal deaths occur.^1^–^2^ There is a need to further facilitate skilled birth assistance and facility-based deliveries (FBDs),^2^–^4^ because skilled birth assistance is the single most important factor in preventing maternal deaths.^5^ Despite its importance, only 20–40% of women in developing countries deliver in a health facility; 6 and approximately 70% of births among poor women take place at home.^7^

The limited use of FBDs is expected to relate to barriers deterring women.^8^–^13^ User fees are one such (financial) barrier discouraging FBD utilisation.^10^–^14^–^16^ Health system factors such as quality also matter.^17^ Although the importance of quality has been confirmed in qualitative study,^18^ few quantitative studies manage to capture the quality of care. Therefore, including such factors, as is the case here, is a necessary addition to the literature.

## Historical perspective

User fees for health services were introduced in many developing countries in the late 1980s, with the aim of financing health care and including maternal health care. Advocates supporting healthcare user fees argue that they enhance the efficient allocation of goods and services by targeting the population in need of the good or service, i.e., low valuation consumers are screened.^19^ Also, if higher prices are perceived to reflect better quality, user fees could increase demand,^20^ a potential virtuous cycle, at least in terms of revenue generation. However, the removal of user fees could also have negative effects on equity and access.^21^–^23^ In an effort to increase healthcare accessibility, by reducing the direct financial cost associated with treatment, most countries in sub-Saharan Africa abolished or reduced user fees for health services, including maternity services and delivery services,^24^–^27^ or exempted certain groups from payment.^28^–^29^

Only limited evidence relating user fee reforms to women's uptake of maternity services is available.^30^–^31^ However, that research generally points to an uptick in FBDs.^28^–^29^–^31^–^32^ Skilled assistance at birth, on the other hand, does not increase following the abolition of user fees.^27^,^33^ This literature has generally ignored the role of supply-side factors,^26^–^34^–^35^ or not accounted for the panel structure of the data.^35^

The context of the policy change in Zambia provides an opportunity to address the preceding concerns. User fees were removed in public health facilities in 54 rural districts in Zambia in April 2006 to improve access to FBDs. Such services include all aspects of preventative and curative services at health posts and health centres, as well as referrals to higher levels.^36^ Prior to the change, antenatal care, family planning and counselling, were exempt from payment, but not delivery services. Health providers were previously allowed to set fees in line with locally defined affordability criteria and varied from ZMK10, 000 ($3) to ZMK30, 000 ($9).^16^,^26^,^34^,^37^ Unsurprisingly, when formal charges are not levied, indirect levies have been. In the absence of user fees in Ghana and Benin, women have been required to purchase supplies — bleach, to sterilise materials used during delivery, gloves and sanitary pads -when admitted to a health facility for delivery.^15^,^38^ Obtaining these supplies could easily delay or prevent the use of delivery services. At the household level, delivery services present additional financial implications, in terms of travel costs, as well as the patient's and the patient's companions' time. Additionally, relatives may have to bring food for the patient, as they wait.^15^

## Background literature

There is an extensive literature relating health financing policy changes to health service utilisation.^24^–^26^,^35^,^39^ Some studies could be biased,^40^ while only a few have accounted for specific time series properties and problems.^24^,^35^ However, in a review of 20 articles, the abolition of user fees has generally been found to have positive effects on the utilisation of health services.^41^

With regard to maternal health services, as would be expected, user fees have had negative effects on utilisation,^14^ while abolition has had the opposite effect.^31^ Although user fees do matter, up to 20 other determinants have been identified.^17^ These are grouped into four broad themes: (1) socio-cultural factors, (2) perceived benefit/need of skilled attendance, (3) economic accessibility and (4) physical accessibility. The identified factors influence decision-making at the individual and household level, but other factors, such as the quality of care, are not easily captured in household surveys. Thus, there is a need to examine the effect of supply-side factors, which is done here.

In additon, the use of ANC services positively affects the utilisation of FBDs and skilled attendance,^10^–^11^,^13^ as does previous delivery at a health facility.^42^–^43^ ANC provides opportunities for health workers to recommend a place of delivery, based on pregnancy risk assessments. Moreover, ANC attendance breeds familiarity with the health system and health facility. However, the positive relationship observed between seeking ANC and delivering at a health facility could result from other confounding factors;^17^,^44^ the same has been suggested for previous deliveries.^17^,^43^ The use of ANC or FBDs may indicate the presence of a nearby health facility offering these services. To address these problems, we include factors to proxy for the availability of health services.

Finally, alternative delivery options should also be considered. In the African context, the primary alternative is a traditional birth attendant (TBA), an alternative that may or may not be an appropriate substitute, due to low levels of literacy, nonexistant to poor training and limited obstetric skills.^45^–^46^ On the other hand, TBAs can be beneficial, especially if they are properly trained.^47^–^48^ However, they need an appropriate support network to work effectively.^16^

## Methods

### Data source

This study uses routine quarterly data, collected within the Health Management and Information System (HMIS) administered by the Ministry of Health (MOH) in Zambia. It contains information on the supply and use of a wide range of health services at all public health facilities nationwide, aggregated to the district. Complete data was available for 46 out of 53 rural districts that abolished user fees in April 2006. However, data from the following districts was discarded: Chibombo, Kapiri Mposhi, Serenje, Chienge, Chavuma, Lukulu, Siavonga and Milenge, because there were multiple missing months of information. From the district level data, we compiled regional data for the 9 provinces from 2003 (q1) to 2008 (q4) (that is T=24 and N=9). Based on the available data and the previously discussed literature, we selected and included six quarterly time series: the proportion of FBDs (ID); average health centre client contacts per day (CC), which measures the staff workload (defined as the total number of patient visits divided by the total number of staff per day); traditional birth attendants per 1000 of the population (TBAs); the proportion of drugs available, based on the percentage of stock-outs of drugs on the essential drug list (DA); the average number of antenatal visits per quarter (ANC); and the population in the province (POP). CC and DA capture the quality of services, while cultural preferences and alternative options are captured by TBAs.

### Model specification

The analysis is founded upon an Interrupted Time Series (ITS) design, complemented by a segmented regression analysis, which is adequate, when only retrospective longitudinal data, before and after an intervention, is available. We disaggregate the data to obtain regional level estimates from Seemingly Unrelated Regressions (SUR), addressing spatial dependence within an error component framework.

Due to the strong persistence observed in FBDs, we specify a dynamic panel model, including one lag of the dependent variable, to assess the impact of the abolition of user fees on FBDs.

**Figure:**
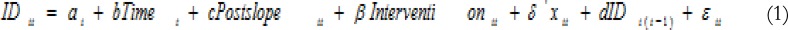


where *ID_it_* is the vector of FBDs in the N=9 provinces; *x_it_* is a vector of explanatory variables and includes time dummies (*t*1, *t*2, *t*3) representing the first, second and third quarters of each year, to account for the cyclicality observed in FBDs, *a_i_* is the regional fixed effect; *Time_t_* is a vector of continuous values indicating time from the start to the end of the study period; *Intervention_it_* is a vector of indicators coded 0 for the pre-invention period and 1 for the post-intervention period; *Postslope_it_* is a vector of indicators coded 0 up to the last point before the intervention and coded sequentially from 1 thereafter; and *ε_it_* is a vector of disturbances.

The analysis accounts for potential cross-sectional dependence. Pooled Ordinary Least Squares (POLS) serves as the baseline; however, Fixed Effects (FE) and Feasible Generalised Least Squares (FGLS) are also considered. FE control for time-invariant omitted variables that differ by province, such as the level of development, health infrastructure and health staff, allowing for intercept heterogeneity; an F-test (Pr>F=0.000) supports the existence of regional fixed effects. The FE method differences out the individual variability across regions. Thus, the FE estimator is a pooled OLS estimator on the demeaned equation (1) and yields unbiased estimates under the assumption of strict exogeniety. In the dynamic specification, the FE estimator with standard errors, robust to moderate levels of cross-sectional dependence in the error term, are implemented.^49^ However, the procedure does not correct for Nickel bias,^50^ an effect that can approach 20%, when T=30.^51^ We implemented a bias-correction procedure suitable for small T and moderate N (10<N<20).^52^ We do not discuss those results, because they are similar to what is obtained without the correction.

The Feasible Generalised Least Squares (FGLS) treats all parameters as random and does not impose any restriction on the error structure, allowing for auto-correlation within panels, cross-section correlation and heteroscedasticity across the units^53^. Furthermore, to link the differences in the policy change within regions to characteristics that vary across regions, we adopt seemingly unrelated regression (SUR).^54^ It is best suited for estimation with cross-section dependence, since it captures the correlation in the error terms across cross-sections, especially when *T > N*.^55^ Therefore, FGLS can be interpreted as pooled SUR, in which estimates represent the average values of the regional coefficients.

## Results

### Preliminary analysis

Panel I of Table 1 contains the means of the data prior to the abolition of user fees (2003(q1)-2006(q1)) to the post-abolition period (2006(q2)-2008(q4)); a statistically significant difference for antenatal visits, drug availability and health centre client contact is uncovered. However, the mean differences do not account for other factors, while the standard errors do not account for within-group correlation. Panel II of Table 1 shows the correlation between the variables. FBDs are statistically significantly correlated with the first lag, suggesting persistence and justifying the dynamic specification in the analysis. ID is positively correlated with ANC; the removal of user fees increases FBDs but lowers the proportion of TBAs. Although the correlation between ID and TBAs is negative and significant, both ID and TBA are significantly correlated to the other regional control variables (ANC, DA, CC and POP). Thus, controlling for these variables will alleviate any reverse causation from ID to TBAs.

**Table 1:** Data summary

		ID	ANC	DA	CC	TBAs	POP$
Panel I: Means and standard errors							
All	Mean	0.355	2.935	0.722	25.728	0.241	11.617
(n=216)	Se	(0.006)	(0.022)	(0.007)	(0.497)	(0.004)	(0.016)
Before	Mean	0.347	3.112	0.745	24.310	0.248	11.570
(n=117)	Se	(0.008)	(0.025)	(0.010)	(0.632)	(0.005)	(0.022)
After	Mean	0.364	2.714	0.696	27.061	0.233	11.672
(n=99)	Se	(0.008)	(0.025)	(0.011)	(0.767)	(0.006)	(0.024)
Diff+	Mean	0.016	−0.386*	−0.049*	3.098*	−0.015*	0.102
	Se	(0.012)	(0.035)	(0.014)	(0.972)	(0.008)	(0.032)

Panel II: Correlation Matrix							

	ID	1					
	ANC	0.219*	1				
	DA	−0.121*	0.095	1			
	CC	−0.032	−0.208*	−0.387*	1		
	TBAs	−0.139*	0.184*	0.503*	−0.217*	1	
	POP	−0.378*	−0.378*	−0.149*	0.442*	−0.397*	
	ID_1	0.830*	0.219*	−0.170*	0.013*	−0.388*	1

Notes: Panel I shows the cross regional means and standard errors of the seven variables. Standard errors are reported in parentheses. Panel II shows the cross regional average correlations among all the variables.

* significance at 5% level.

+ Diff gives the difference between before and after (After-before).

$ Population has been transformed to log values.

### Diagnostic tests

To determine the appropriate methodology to be applied, we first ascertained the properties of the data. We implemented a series of panel data tests to uncover panel unit roots and deal with the possibility of region-specific trends.^56^–^57^ Furthermore, FBDs in one region may be affected by not only a local change in fees charged (due to the abolition of user in the region) but also by similar changes occurring in other regions. In the presence of cross-sectional dependence, ignoring it would result in inefficient estimates and misleading inference, especially if the source of the cross-section dependence is correlated with the regressors.^55^ Therefore, we also considered a test for cross-sectional dependence that is robust to non-stationarity, parameter heterogeneity and performs well, even in small samples.^58^ Table 2 reports the average pair-wise correlation coefficient, and the CD test statistic for all the variables, in levels. The results indicate the presence of cross-section correlation between the provinces for all the variables.

**Table 2:** Various Tests of Panel Data Properties

	Breitung t-stat		(III)IPS Wtbar		Cross-Section Dependence	
	Without trend	With trend	Without trend	With trend		CD test
ID	−2.778*	−3.414*	−3.320*	−3.774*	0.331	9.72*
ANC	0.035	−3.535*	−1.547	−3.777*	0.731	21.49*
CC	−4.204*	−4.212*	−3.090*	−4.380*	0.382	11.24*
TBAs	−2.890*	0.228	−2.367*	−2.882*	0.156	4.58*
DA	−2.896*	−1.621*	−2.761*	−3.341*	0.229	6.74*
POP	1.567	−2.672*	−0.607	−4.812*	0.998	29.32*
ID_1	−2.685*	−1.878*	−3.237*	−3.690*	0.367	10.56*

Breitung and Im Pesaran and Shin (IPS) unit root test statistics along with and Pesaran (2004) CD test reported.

* significant at 5% level.

Table 2 also presents two panel unit root test results, with and without trend. Both tests reject the null of a panel unit root for all the variables, except antenatal visits without a trend. Given cross-section dependence, we augment these unit root tests cross-section averages, taking into account cross-sectional dependence, due to worries over test size.^59^ Fortunately, the results obtained were similar to the unit root tests; thus, they are not reported here. We conclude that all the variables are trend stationary, and proceed accordingly.

### Policy change at aggregate level

Results of the static and dynamic representations for the three estimators are presented in Table 3. Panel I contains results from a simple model with only ITS controls. Panel II, on the other hand, includes additional controls, but focuses on the static representation. Finally, panel III contains results from the dynamic model, which includes one lag of the dependent variable; thus, only FE and FGLS results are meaningful, while POLS, FE and FGLS are meaningful for the other models.

**Table 3:** Estimation results: OLS, FE, RC, and FGLS estimates of the impact of the abolition of user fees on facility-based deliveries

	(I) Indicators only			(II)Static			(III) Dynamic		
ID	POLS	FE	FGLS	POLS	FE	FGLS	FE	FGLS
Intercept	−0.014	−0.014	0.001	−0.004	−0.006	−0.003	0.009	0.012**
Postslope	0.005	0.005**	0.006**	0.004***	0.005***	0.005**	0.004**	0.001
Time	−0.001	−0.001	−0.001	0.002**	−0.008	0.002**	−0.007***	−0.000
	−0.020	−0.020**	− 0.020***	−0.009	−0.039	−0.013***	−0.015*	0.010**
	−0.014	−0.014**	− 0.014***	−0.010	−0.029	−0.011***	0.001	0.030***
	0.035*	0.035***	0.035***	0.029***	0.019	0.032***	0.047***	0.072***
ANC				0.050***	0.048***	0.038***	0.015	0.013
DA				0.156***	0.182***	0.067**	0.143***	0.039**
CC				−0.002	−0.002	0.000	−0.002**	0.000
TBAs				−0.096	−0.077	−0.048*	−0.003	−0.035*
POP				−0.048	1.149	−0.152***	0.797***	−0.022*
FBD-lag							0.470***	0.848***
Constant	0.354***	0.354***	0.342***	0.711	−13.062	1.940***	−9.088***	0.224
F-test (Pr>F)	0.017	0.000		0.000			0.000	
Wald test (Pr>Chi2)			0.000			0.000		0.000

*** p<0.01,

** p<0.05,

* p<0.1;

standard errors not reported.

Because the assumptions associated with each set of models in each of the panels differ, it is necessary to whittle down the results to the one result or set of results that is most plausible. Within I, where all of the explanatory variables are based on the ITS design, the assumptions are: (i) there is no persistence in the dependent variable and (ii) no other variables influence FBDs, other than the policy. Within II, assumption (ii) is relaxed, and, within III, both (i) and (ii) are relaxed. Persistence in the dependent variable leads to a preference for results in III, rather than results in either I or II. Additionally, recall that POLS is underpinned by homogeneity of all effects across regions and time, and, therefore, is not valid in a dynamic setting, while FE allows for time-invariant regional differentiation and FGLS allows for unspecified correlation in the errors. In this setting, both auto-correlation (Pr>F=0.0045) and hetero-scedasticity (Pr>Chi2=0.000) are present in the data.^60^–^61^ Therefore, the FGLS specification is generally preferred. In other words, the results in the last column represent the preferred results.

Therefore, based on the FGLS estimates, we conclude that there was an immediate 1.2 percentage point increase, or 3.4% increase in FBDs relative to baseline, following the policy change. However, no statistically significant increase in the trend in FBDs could be identified, which means that FBDs did not continue to rise after the immediate increase. However, the lagged dependant variable is positive and statistically significant, suggesting that previous deliveries in the facility affect current deliveries, a result that is consistent with previous work.^42^,^62^ Drug availability (DA), which proxies for the quality of services, is positively associated with FBDs, whilst the presence of TBAs is negatively associated with FBDs.^17^ ANC visits, however, are not significantly associated with FBDs and the (lack of) result differs from previous analyses. 10 Provincial-level population growth has a negative and statistically significant impact on FBDs, which is expected, since population growth can strain the provision of health services, especially if the supply of healthcare inputs remains fixed.

### Policy change across provinces

Although the preceding results imply that the elimination of user fees for delivery services increased the use of facility-based delivery services, at least at the national level, that analysis could mask heterogeneity. We turn to this consideration, through the estimation of a SUR model. The results of the analysis are presented in Table 4. Panel I contains region-specific regression results. In addition to the SUR estimates, we also report the LM test of spatial independence.^63^ If rejected, the SUR estimator improves the efficiency of the region-specific estimates, through the incorporation of cross-equation residual correlation. Panel II, in Table 4, describes the degree of correlation between those residuals.

**Table 4:** Seemingly unrelated regression estimates: Provincial level

	Central	Copperbelt	Eastern	Luapula	Lusaka	Northern	N/Western	Southern	Western
**Panel I: Provincial estimates of the impact of the abolition of user fees on facility-based deliveries**									
Inter	0.044	−0.083***	−0.004	0.003	−0.031	−0.017	−0.003	0.018	−0.047**
Postslope	−0.007**	0.012***	0.004	0.006***	0.020***	0.001	0.006	−0.001	0.019***
Time	−0.000	−0.011***	0.005**	−0.001	0.000	0.002	0.002	−0.002	−0.003
t1	0.016	−0.072***	−0.011	−0.004	0.036**	−0.001	−0.013	0.001	−0.010
t2	0.016	−0.073***	0.015	0.014	0.041**	0.011	−0.008	0.006	0.010
t3	0.065***	0.008	0.065***	0.033***	0.021	0.028***	0.054**	0.028***	0.054***
ANC	−0.043	−0.063***	0.052	0.006	0.032	0.021	0.055	−0.100**	0.052*
DA	0.067	−0.248***	0.175**	0.217***	0.116	−0.005	0.129	0.185***	0.474***
CC	−0.001	−0.001	−0.003***	−0.006***	−0.006**	0.000	−0.004*	−0.006***	0.004*
TBAs	−0.100	−0.153***	0.264***	0.171***	0.352***	0.032	0.032	0.045	0.159
Lpop	0.024	0.115***	−0.019	0.012*	−0.023	0.016	0.026	0.041***	−0.020***
ID_1	0.500***	−0.341***	0.474***	0.391**	0.707***	−0.061	0.059	0.214	−0.096
Breush_Pagan test of independence: chi2(36) = 54.464, Pr = 0.0249									

**Panel II: Correlation Matrix of residuals**									

Central	1								
Copperbelt	0.431	1							
Eastern	−0.0862	0.0727	1						
Luapula	0.1857	0.4216	0.0954	1					
Lusaka	0.0786	−0.2938	0.3061	−0.2045	1				
Northern	0.0156	0.4126	−0.1628	0.1367	−0.2732	1			
N/Western	−0.2096	0.3159	0.1979	0.3097	0.0121	−0.1191	1		
Southern	0.2738	0.4985	−0.0193	0.2414	−0.2064	0.3901	0.041	1	
Western	0.1398	0.3188	−0.4521	−0.2136	0.2000	0.3318	−0.1129	0.1505	1

*** p<0.01,

** p<0.05,

* p<0.1.

Panel I shows the SUR estimates for each province. Panel II shows the cross-provincial correlation matrix

As might be expected in any country, and possibly moreso in a developing country, policy impacts are not estimated to be the same across all regions. The results in Panel I suggest that the abolition of user fees led to a statistically significant and immediate negative reduction in FBDs in two provinces. In the Copperbelt and Western provinces, that reduction was 8.3 percentage points (17% with reference to the baseline) and 4.7 percentage points (13% with reference to the baseline) respectively. However, post-intervention, there was a trend increase in FBDs, quarter-on-quarter, in four of the nine provinces (ranging from 1.9% in Luapula to 6.8% in Lusaka), although Central province experienced a relatively small, but statistically significant, decrease in FBDs (0.7 percentage point (2.3%) overtime. In addition to the previously uncovered differences across regions, cross-sectional independence is rejected (Pr=0.0249).

Although the reduction in FBDs is unexpected, one can speculate that the reduction in user fees could have increased the utilisation of other health services, which, in turn, had a negative effect on FBDs. For instance, the Copperbelt province is a relatively urban province and a hub for health care professionals; therefore, individuals from the surrounding regions often travel to the province to seek care. In fact, provincial level correlations between the residuals, described in the correlation matrix presented in Panel II, indicate a relatively high degree of correlation between the Copperbelt and other regions. It might also have been expected that provinces close to each other would exhibit relatively larger degrees of dependence; that is not entirely true. For instance, about 5 provinces, namely, Luapula, Northern, NorthWestern, Southern and Western provinces have a relatively high positive correlation with the Copperbelt province, but not necessarily with regions that are close to them. Potentially, there is spatial auto-correlation, but we leave that for future research.

Since user fees are not the only factors determining facility delivery, and, therefore, the relative importance of fees relative to other barriers (such as quality of care) is likely to vary from province to province, these results are not unexpected. Furthermore, some provinces may have greater capacity to deal with an increase in utilisation, and, thus, maintain the quality of care provided. In support of the previous hypothesis, there is evidence that the drugs and financing that were meant to be provided, ostensibly to help health centres deal with the hoped-for influx in deliveries, were not successfully delivered to all districts and facilities.^34^,^64^

## Discussion

The abolition of user fees reduces the financial cost of treatment, and is expected to increase utilisation rates. Studies in Zambia and other developing countries have uncovered increases in the use of health services by some population groups, after the removal of user fees,^24^–^26^,^35^ and our results at the national level are consistent with that in literature. Moreover, the findings highlight the importance of quality of services in encouraging FBDs at the national level, but there are important variations at the regional level.

The national level findings suggest that TBAs statistically significantly reduce FBDs. At the regional level, a similar result is found for the Copperbelt province, while in regions such as the Eastern, Luapula and Lusaka provinces the association is positive. The positive association suggests that harnessing TBA potential is worth additional investigation. Within Zambia, the involvement of trained TBAs in the delivery process remains an important strategy, particularly in rural areas, where health worker scarcity is a problem.^16^ Furthermore, trained TBAs can reduce perinatal deaths, neonatal deaths and stillbirths,^47^–^48^ although others argue that TBAs offer poor obstetric services.^45^–^46^ Between 1970 and 1990, the World Health Organisation promoted TBA training, as one strategy to reduce maternal and neonatal mortality; however, there is insufficient evidence to establish the potential for TBA training to improve peri-neonatal mortality.^65^ Given that a larger share of women in Zambia were assisted by TBAs in 2007 (23.5%) compared to 2001/2 (11.7%),^66^ the role of TBAs cannot be ignored. Similarly, the scarcity of health personnel in low-income countries, especially personnel focussed on women's health, means that non-FBDs will continue to play a significant role in health service provision.^6^ Thus, reducing maternal mortality may require the implementation of interventions, which are country-specific and include TBAs, due to differences in the local contexts.

Additionally, although user fees were not a significant source of revenue to the health facilities, due to routine costs, they allowed health facilities in rural areas to support TBAs. For example, user fees were often redistributed to TBAs, in the form of tokens of appreciation, to encourage women to deliver at health facilities.^34^ Fees were also used to purchase cleaning agents (bleach) and food for inpatients.^34^ However, with the abolition of user fees, TBA support has been significantly reduced. Moreover, following the abolition of fees in Ghana, Benin and Zambia, women are reportedly required to bring bleach, gloves and syringes with them, when delivering at a health facility.^15^,^64^Additional requirements, such as these, act as a barrier to the utilisation of delivery services, and may even exceed the levels of the abolished user fees. Such barriers are problematic. In comparison, TBA delivery costs are reportedly affordable, especially for the poor, because the payments are negotiable, both in amount and timing, while in-kind payments are also accepted.^8^

## Conclusion

This research investigates the impact of user fee abolition, with specific focus on regional variation, on FBDs using a panel of 9 Zambian provinces covering the period 2003(q1) to 2008(q4). Different models are estimated to address heterogeneity, auto-correlation, hetero-scedasticity and cross-section dependence within a panel data context. After the econometric issues were addressed, the aggregate results provide strong evidence that the abolition of user fees had an immediate positive impact on FBDs. However, the increase was not sustained via an increase in the trend, and the increase was economically small, 1.2 percentage points or 3.4% or pre-intervention births. The aforementioned aggregate increase could not be ascribed to an increase in any particular region. Instead, immediate decreases were uncovered in some regions, while trend increases were uncovered in other regions. In other words, the aggregate results mask interesting regional heterogeneity. From a policy perspective, that heterogeneity is likely to be important; motivations for non-FBDs might be social or cultural, factors not easily altered through reductions in the direct cost of delivery. Similary, although it was not possible to consider indirect charges, as data was not available, anecdotal evidence suggests that facilities have followed a cost-shifting strategy, and that strategy could account for the economically small user fee impacts estimated here, as well as the variation in the effects uncovered.

In addition to user fee effects, the analysis also identified a TBA impact and a quality of service impact. At the aggregate level TBAs are associated with a reduction in FBDs; however, at the regional level, the impact was more varied. In some provinces, the association was positive, while in others it was negative. With respect to service quality, a strong positive impact was uncovered at the national level, a result that carried over to a number of provinces. Again, the aggregate results masked interesting regional heterogeneity.

Although the conclusions are reasonably general, there are some limitations worthy of further analysis. For example, the routine data used in the analysis could either be too sporadic or too frequent. Quarterly data provides less data than monthly data, and might hide trends in utilisation; however, both quarterly data and monthly data might also be too noisy for the identification of trends. Another concern is that routine data neither provides information about non-users nor provides socio-economic and related characteristics of users. Specific information about users and non-users is potentially useful in exploring response heterogeneity, user fee abolition may have affected poor women differently than non-poor women. Unfortunately, the observed increase could reflect better recording, rather than actual increases in FBDs. Additionally, the routine data only covered public health facilities in Zambia. Thus, the observed changes in utilisation might not be a reflection of all health facilities in the country, since most private health facilities are not, yet, incorporated into the HMIS.

Future research, which is likely to complement this research, should seek to link individual-level data, from household surveys, to facility-level data. Such a link between the supply and demand sides of the market could underpin an analysis of the impact of the policy change on maternal health-seeking behaviour. Recent Demographic and Health Survey data include geographic positioning system (GPS) information, which can be used to tie sample clusters to routine data. Data tied together in this fashion could be used for the analysis of mother's health or child health, relating user fee abolition policies to maternal health outcomes and, subsequently, to child health outcomes. Finally, future research could consider modelling other types of spatial correlation which gives more importance to the distance between regions.
